# Successes and gaps in the HIV cascade of care of a high HIV prevalence setting in Zimbabwe: a population‐based survey

**DOI:** 10.1002/jia2.25613

**Published:** 2020-09-24

**Authors:** Nolwenn Conan, Rebecca M Coulborn, Erica Simons, Abraham Mapfumo, Tsitsi Apollo, Daniela B Garone, Esther C Casas, Adrian J Puren, Menard L Chihana, David Maman

**Affiliations:** ^1^ Epicentre Paris France; ^2^ Médecins Sans Frontières (MSF) Harare Zimbabwe; ^3^ Ministry of Health and Child Care Harare Zimbabwe; ^4^ Southern Africa Medical Unit MSF Cape Town South Africa; ^5^ National Institute for Communicable Diseases (NICD) National Health Laboratory Service Johannesburg South Africa; ^6^ Division of Virology, School of Pathology University of the Witwatersrand Medical School Johannesburg South Africa

**Keywords:** HIV, cascade of care, incidence, prevalence, ART, viral suppression

## Abstract

**Introduction:**

Gutu, a rural district in Zimbabwe, has been implementing comprehensive HIV care with the support of Médecins Sans Frontières (MSF) since 2011, decentralizing testing and treatment services to all rural healthcare facilities. We evaluated HIV prevalence, incidence and the cascade of care, in Gutu District five years after MSF began its activities.

**Methods:**

A cross‐sectional study was implemented between September and December 2016. Using multistage cluster sampling, individuals aged ≥15 years living in the selected households were eligible. Individuals who agreed to participate were interviewed and tested for HIV at home. All participants who tested HIV‐positive had their HIV‐RNA viral load (VL) measured, regardless of their antiretroviral therapy (ART) status, and those not on ART with HIV‐RNA VL ≥ 1000 copies/mL had Limiting‐Antigen‐Avidity EIA Assay for cross‐sectional estimation of population‐level HIV incidence.

**Results:**

Among 5439 eligible adults ≥15 years old, 89.0% of adults were included in the study and accepted an HIV test. The overall prevalence was 13.6% (95%: Confidence Interval (CI): 12.6 to 14.5). Overall HIV‐positive status awareness was 87.4% (95% CI: 84.7 to 89.8), linkage to care 85.5% (95% CI: 82.5 to 88.0) and participants in care 83.8% (95% CI: 80.7 to 86.4). ART coverage among HIV‐positive participants was 83.0% (95% CI: 80.0 to 85.7). Overall, 71.6% (95% CI 68.0 to 75.0) of HIV‐infected participants had a HIV‐RNA VL < 1000 copies/mL. Women achieved higher outcomes than men in the five stages of the cascade of care. Viral Load Suppression (VLS) among participants on ART was 83.2% (95% CI: 79.7 to 86.2) and was not statistically different between women and men (*p* = 0.98). The overall HIV incidence was estimated at 0.35% (95% CI 0.00 to 0.70) equivalent to 35 new cases/10,000 person‐years.

**Conclusions:**

Our study provides population‐level evidence that achievement of HIV cascade of care coverage overall and among women is feasible in a context with broad access to services and implementation of a decentralized model of care. However, the VLS was relatively low even among participants on ART. Quality care remains the most critical gap in the cascade of care to further reduce mortality and HIV transmission.

## 1 INTRODUCTION

Zimbabwe faces a generalized HIV epidemic [1]. An estimated 1.2 million Zimbabweans aged 15 to 64 years old live with HIV, representing an HIV prevalence in this age group of 14.1% according to the 2015 to 2016 Zimbabwe Population‐Based HIV Impact Assessment (ZIMPHIA) [1]. In 2016, the government of Zimbabwe adopted universal antiretroviral therapy (ART) initiation regardless of CD4 count (“Treat all”), as recommended by the World Health Organization (WHO) [2, 3]. In 2014, the Joint United Nations Programme on HIV/AIDS (UNAIDS) endorsed the 90‐90‐90 targets as key indicators against which to measure progress in the global HIV response: by 2020, 90% of all people living with HIV would know their HIV status (first 90), 90% of people aware of their HIV‐positive status would receive sustained ART (second 90) and 90% of all people receiving ART would have a viral load suppression (VLS) (third 90) [4]. National ZIMPHIA findings showed that, in Zimbabwe, 74.2% of HIV infected participants aged 15 to 64 years reported knowing their HIV status, 86.8% of those who knew their status were on ART and 86.5% of those on ART were virally suppressed [1].

Médecins Sans Frontières (MSF) has been working in Zimbabwe since 2002, mostly in support of HIV/AIDS programmes, in collaboration with the Ministry of Health and Child Care’s (MOHCC). In January 2011, MSF opened a project to improve HIV service delivery in Gutu District, Masvingo Province, with a population of 203,533 persons [5]. The project aimed to decentralize ART delivery for the first time in the district through task‐shifting from doctors (hospital level) to nurses (primary health facility level), clinical mentorship to nurses, establishment of pre‐packaged drug pick‐up points, supply donation, stock out management, enhancement of laboratory services and routine HIV‐RNA viral load (VL) monitoring for all clients on ART. The gradual implementation and scale‐up of activities later resulted in the creation of community ART refill groups (CARGs), which consisted of groups of HIV‐positive individuals in which members alternated clinical visits and pick‐up of ART drug refills at the clinic for distribution to fellow members of the community group [6].

The cascade of care constitutes a critical means by which to assess programme effectiveness. This population‐based survey assesses HIV prevalence, incidence and programme effectiveness along each stage of the cascade of care more than half a decade after MSF began its activities in Gutu District.

## 2 METHODS

### 2.1 Study design

We conducted a cross‐sectional study between September and December 2016, targeting both males and females ≥15 years of age. We used two‐stage cluster sampling (first stage = enumeration area (EA); second stage = households) with a probability proportional to population size to select study participants. In the first stage we selected 113 clusters from the 440 EAs in Gutu mapped by the Zimbabwe National Statistic Agency [5]. Then an equal number of households (25 dwellings per EA) was randomly selected using point‐based spatial sampling from a list of points. Dwellings which were vacant, destroyed or not found were replaced by a reserve list of points. All residents of the study area and visitors who had spent at least the previous night in the selected household were eligible for the study. Eligible occupants refusing participation in the study were not replaced.

### 2.2 Data collection and laboratory procedures

Individual face‐to‐face interviews with participants provided information on demographic characteristics, HIV testing habits and ART intake if previously tested.

**Figure 1:**
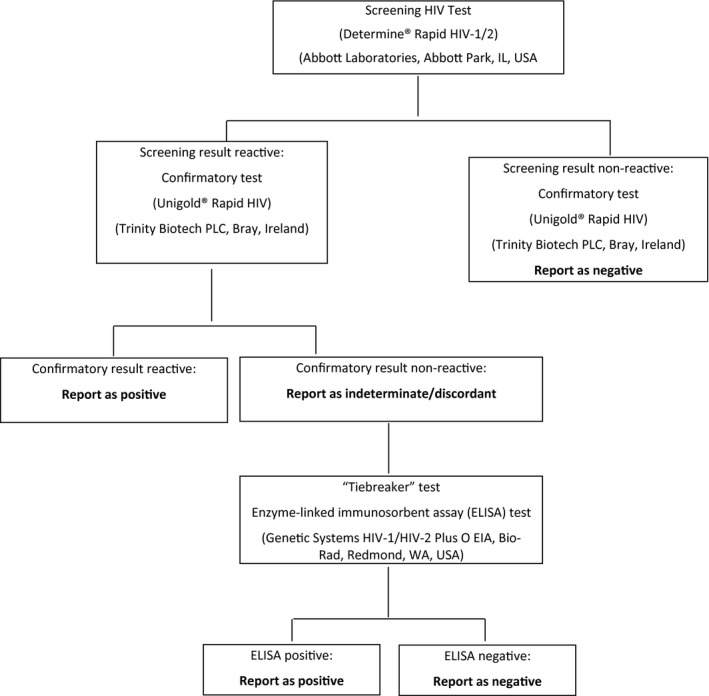
HIV testing algorithm, HIV Counselling and Testing Policy guidelines in Zimbabwe.

HIV testing was proposed to all study participants by a certified counsellor [7, 8], irrespective of previous knowledge of HIV status. The serial algorithm is presented in Figure 1.

From participants who tested HIV‐positive, venous blood samples were collected for additional testing: 1) VL testing on all participants using Dried Blood Sport (DBS) (Nuclisens HIV‐1 QT assay BioMerieux®, Marcy‐Etiole, Rhône), with a limit of detection of 802 copies/mL. For participants with a VL between 1000 and 5000 copies/mL, reassessment of HIV‐RNA VL on plasma was performed using the same platform; 2) ART detection was conducted on DBS for participants who declared not to be on ART: A Liquid Chromatography (LC) coupled to Tandem Mass Spectrometry (MS/MS) qualitative assay [9] assessed the three main ART (nevirapine, efavirenz and atanazavir) as a proxy for all ART in use in the public sector of Zimbabwe. The estimated time post ingestion to the quantifiable level of 0.02 µg/mL was 12 to 28 days for efavirenz, eight to nine days for nevarapine and 2.5 days for atanazavir. As such, a positive result was expected if the last dose was taken as prescribed and 3) recent infection was tested using the single‐well limiting antigen avidity EIA (LAg‐Avidity EIA) assay with a HIV‐RNA VL ≥ 1000 copies/mL, as described in detail by Duong *et al*. [10], on participants not on ART (confirmed by the LC‐MS/MS assay). Participants with an optic density <1.5 were considered recently infected. The overall mean duration of recent infection in our algorithm was 130 days (95% Confidence Interval (CI): 118 to 142). A false recent rate estimated at <0.0001% was used to account for misclassification. HIV incidence was estimated cross‐sectionally according to the method of Kassanjee *et al*. [11].

### 2.3 Definitions

HIV testing coverage was defined as the proportion of study participants who received at least one HIV test any time prior to the survey. Classification of participants according to their stage along the HIV cascade of care relied on self‐reporting and laboratory data [12]. We calculated the five stages of the cascade of care among all HIV‐positive participants as follows: HIV‐positive status awareness (Stage one) was defined as a history of at least one positive HIV test prior to the study. Linkage to care (Stage two) was defined as at least one medical consultation for HIV care after an HIV‐positive test result received prior to the study. Being in care (Stage three) was defined as still receiving HIV‐related medical consultations at the time of the study. ART use (Stage four) was established from the individual health notebook for participants reporting to be on ART and from a positive blood ART detection test for participants reporting not to be ART. Those who lacked an ART detection test result were classified as not on ART. Those with a positive ART detection test result were reclassified as on ART, on care, linked to care and aware of their HIV‐positive status. Viral load suppression (VLS) (Stage five) was defined as an HIV‐RNA VL < 1000 copies/mL among all HIV‐positive participants. UNAIDS targets were also calculated.

### 2.4 Data analysis

Data were captured from paper‐based questionnaires, laboratory information management systems and registers, according to the standard procedures of each laboratory. Questionnaire data were double entered into EpiData 3.1 (EpiData Association, Odense, Denmark). Statistical analyses were performed with STATA 15 (StataCorp, College Station, TX, USA). Descriptive analyses were weighted to account for the selection probability by the cluster sampling procedure. In order to compare with the national survey [1], subanalyses were also performed on study participants 15 to 64 years of age. Outcomes were stratified by sex and reported with corresponding 95% CI. Categorical variables were compared using Pearson chi‐square or fisher exact test, as appropriate, and proportion with z‐proportion test. *p*‐values below 0.05 were considered statistically significant.

### 2.5 Ethical approvals

The Medical Research Council of Zimbabwe (Reference: MRCZ/A/2075) and the MSF Ethic Review Board (Reference: ID1619) approved the study. Study materials were available in both English and Shona. Prior to participation, researchers obtained written informed consent from adult participants and, in the case of minors aged less than 16, from a parent/guardian, with assent from the minors.

## 3 RESULTS

### 3.1 Demographics

**Figure 2:**
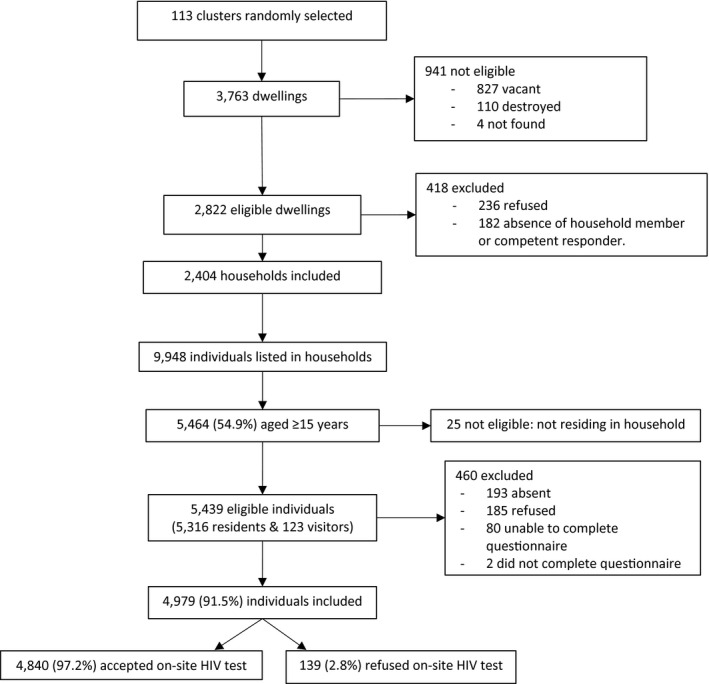
Flow chart of eligibility and inclusion of study participants, Gutu District, Zimbabwe 2016.

Out of 113 clusters and 2404 households, 5439 individuals were eligible and 4979 (91.5%) included in the study, with 2933 (58.9%) female participants. The inclusion rate was higher among women than men (94.0% vs. 88.3%; *p* < 0.001). Out of 4979 participants, 4840 (97.2%) accepted to be tested (Figure 2).

The median age of study participants was 39 years (Interquartile range [IQR]: 23 to 58). More than 90% of participants had lived in Gutu District for more than five years prior to the survey.

#### HIV testing coverage

Overall, 73.5% (95% CI: 72.3 to 74.7) of participants had received at least one HIV test prior to the survey. HIV testing coverage was higher among women than among men: 78.7% versus 66.1%; *p* < 0.001. The 1319 untested participants were predominantly men (52.6%), and aged less than 30 years old (40.0%).

HIV testing coverage among HIV‐negative participants was also relatively high with 70.5% (95% CI: 69.1 to 71.9) who received an HIV test prior to the survey, 76.1% among women and 62.6% among men (*p* < 0.001).

Among the 2885 HIV‐negative participants already tested for HIV and who reported their most recent HIV testing date, 57.4% (95% CI: 55.6 to 59.2) were tested one year or less prior to the survey.

#### HIV prevalence and incidence

**Figure 3:**
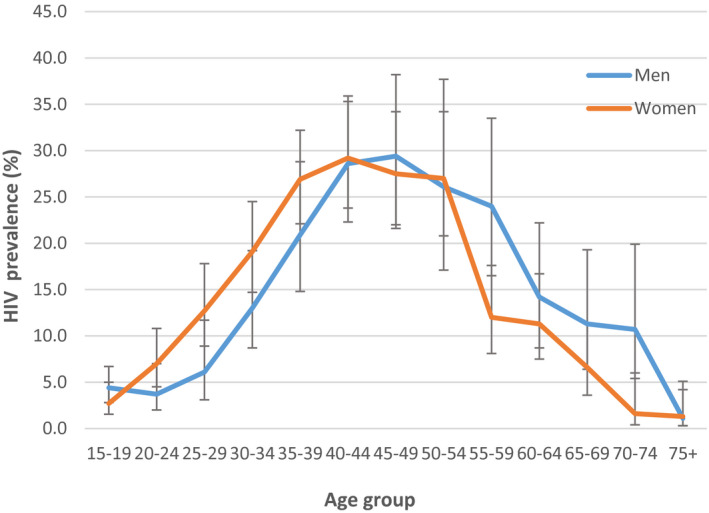
HIV prevalence by five‐year age group among men and women, Gutu District, Zimbabwe 2016.

Among the 4840 participants who accepted an HIV rapid test, 655 participants were HIV‐positive. The weighted HIV prevalence was 13.6% (95% CI, 12.6 to 14.5) overall, and 15.4% (95% CI, 14.3 to 16.5) when restricting analysis to participants 15 to 64 years old. HIV prevalence was higher among women compared to men: 14.4% versus 12.3%; *p* = 0.034. For both women and men, prevalence was lower in the youngest and oldest age groups. The HIV prevalence peaked at 40 to 44 years among women and 45 to 49 years among men (Figure 3). The median age of HIV‐positive participants was 41 years [IQR: 34 to 50] for women and 43.5 years [IQR: 36 to 54] for men.

Of the HIV‐positive participants, five were classified as recently infected using the LAg‐Avidity EIA algorithm (3 women and 2 men). Overall HIV incidence was estimated at 0.35% (95% CI: 0.00 to 0.70), equivalent to 35 new cases/10,000 person‐years in the district. Incidence was similar in women and men (36 new cases/10,000 person‐years (95% CI: 0 to 81) versus 34 new cases/10,000 person‐years (95% CI: 0 to 83)). When restricted to 15‐ to 64‐year‐olds, overall HIV incidence was 0.43% (95% CI: 0.00 to 0.86).

### 3.2 Cascade of care among HIV‐positive participants

Among the 118 participants self‐reporting non‐use of ART, 7.6% tested positive for ART and were reclassified as on treatment, on care, linked to care and aware of their HIV‐positive status.

The overall HIV‐positive awareness among HIV‐positive participants was 87.4% (95% CI: 84.7 to 89.8), 85.5% (95% CI: 82.5 to 88.0) of the participants were linked to care and 83.8% (95% CI: 80.7 to 86.4) were in care (Table 1). Among the 82 participants unaware of their HIV‐positive status, 17.1% (95% CI: 10.3 to 27.0) had received their most recent HIV test less than 6 months prior to the survey and 35.4% (95% CI: 25.6 to 46.5) had never been tested.

**Table 1:** Steps of the HIV cascade of care by sex among HIV‐ participants, Gutu District, Zimbabwe 2016

	Overall		Women		Men	
	n/N	% (95% CI)	n/N	% (95% CI)	n/N	% (95% CI)
Aware on HIV‐positive status	571/653	87.4 (84.7 to 89.8)	377/410	92.0 (88.9 to 94.2)	194/243	79.8 (74.3 to 84.4)
Linked to care	558/653	85.5 (82.5 to 88.0)	370/410	90.2 (87.0 to 92.8)	188/243	77.4 (71.7 to 82.2)
In care	547/653	83.8 (80.7 to 86.4)	365/410	89.0 (85.6 to 91.7)	182/243	74.9 (69.1 to 80.0)
On ART	543/653	83.0 ( 80.0 to 85.7)	364/410	88.8 (85.3 to 91.5)	179/243	73.4 ( 67.5 to 78.5)
Viral load < 1000 copies/mL	447/624	71.6 ( 68.0 to 75.0)	296/393	75.3 (70.8 to 79.3)	151/231	65.4 (59.0 to 71.2)

Overall, 83.0% (95% CI: 80.0 to 85.7) of the HIV‐positive participants were on ART. The median time since ART initiation was 51.4 months [IQR: 28.7 to 73.3] (four years and three months). The proportion of participants who presented an HIV‐RNA VL < 1000 copies/mL (virologically suppressed) was 71.6% (95% CI: 68.0 to 75.0). Out of the 177 unsuppressed participants (HIV‐RNA VL ≥ 1000 copies/mL), 54.8% (95% CI: 47.4 to 62.1) were women and 39.0% (95% CI: 32.0 to 46.4) were aged 30 to 44 years. Of the 175 unsuppressed participants with complete information on HIV‐positive status awareness and ART coverage, 50.3% (95% CI: 42.9 to 57.1) were on ART, while 37.7% (95% CI: 30.8 to 45.2) were unaware of their HIV‐positive status and 12.0% (95% CI: 7.9 to 17.8) were aware of their HIV‐positive status, but not on ART. All stages of the cascade of care were associated with sex, with the highest coverages among women. A breakdown of the HIV cascade of care by sex is presented Table 1.

### 3.3 90‐90‐90‐ targets

**Figure 4:**
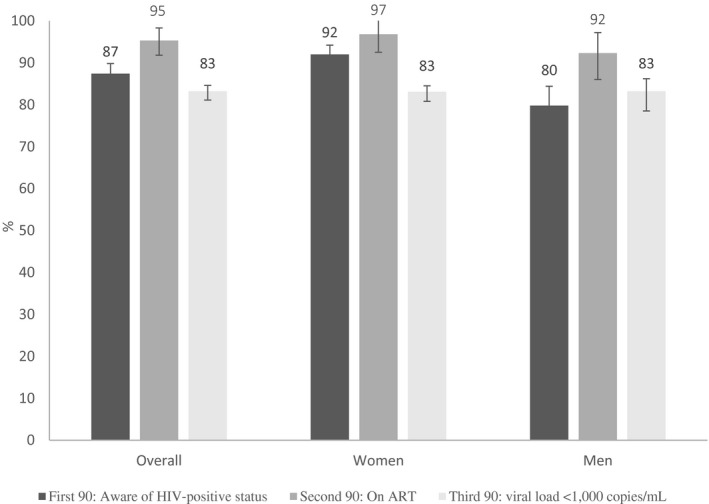
90‐90‐90‐UNAIDs target among participants ages 15 years and more, by sex, Gutu District, Zimbabwe 2016.

We also calculated the 90‐90‐90 target among the participants aged 15 years and older (Figure 4). Overall, HIV‐positive status awareness (first 90) was 87.4% (95% CI: 84.7 to 89.8), ART coverage among participants aware of their status (second 90) was 95.3% (95% CI: 93.2 to 96.7) and VLS among those on ART (third 90) was 83.2% (95% CI: 79.7 to 86.2). While women were more likely to be on ART than men (*p* < 0.01), they achieved similar VLS to men once on ART (*p* = 0.98).

Among the 482 participants who reported being on ART for more than six months and who accepted a VL test, 83.8% (95% CI: 80.2 to 86.9) were virally suppressed and the proportion of VLS did not differ according to the duration on ART beyond six months.

When restricted to 15‐ to 64‐year‐olds, 88.0% (95% CI: 85.2 to 90.4) of HIV‐positive participants knew their status, 95.2% (93.0 to 96.7) of those were on ART, and 83.3% (95% CI: 79.8 to 86.3) of those were virologically suppressed.

## 4 DISCUSSION

We found that coverage results of the five HIV cascade of care stages were high among HIV‐positive participants living in a high prevalence setting, suggesting that the HIV programmes in the district have been effective, particularly for women. Incidence results were relatively low, probably due to the promising results of the 90‐90‐90 targets overall and among women after five years of implementation of decentralised HIV activities. Despite similar results among women and men in VLS once on ART, men were less diagnosed and on ART than women. A meaningful proportion of participants, especially men, had not been tested for HIV before.

To our knowledge, this is the first study to assess the HIV prevalence, incidence and cascade of care at district level in Zimbabwe. HIV prevalence in Gutu was similar to the national [1]. Similar to other settings [13, 14, 15], HIV prevalence was higher among women than men. Despite residing in a high HIV‐prevalence setting, more than a quarter of participants and almost one third of men had never been tested for HIV before. One possible explanation could be that the time, cost and distance barriers associated with facility‐based voluntary counselling and testing (VCT) may hinder men who represent the main breadwinners in the population [16, 17, 18, 19, 20, 21]. Targeting men through oral self‐testing, integration of VCT at or close to the workplace, and in the community may improve access to HIV testing and subsequently increase HIV status awareness [16, 17, 18, 19, 20, 21, 22, 23, 24]. In addition, approximately 17% of participants who tested positive during the survey had received a negative HIV test result less than six months prior to the survey. This suggests that those participants may have important high‐risk factors, including false reassurance from previous negative test results. Counselling individuals who test negative on strategies to remain negative appear especially pertinent in this setting.

HIV‐positive status awareness was higher in our survey compared to the national survey (88% vs. 73%) [1], and to other population‐based surveys from Malawi, Kenya, Swaziland and South Africa [13, 14, 15, 25, 26, 27]. Although difficult to compare the results directly, implementation of HIV testing in the facilities [28], as well as night‐time HIV testing clinics and outreach testing provided by MSF and HIV campaigns at various hot spots in the district could help to explain this success. Additionally, MSF has engaged focus group discussion with community leaders and religious objectors since 2011 that may have helped to dispel the stigma around HIV testing and boosted HIV testing rates. Consistent with some of these studies [14, 25, 27], men were less likely to be aware of their HIV status than women.

The proportion of HIV‐positive participants linked to care and in care was high. Decentralization of service delivery, engagement with community leaders to link the community with the healthcare facility and scale‐up of CARGs over the last five years may have contributed to the high ART coverage among individuals who were aware of their HIV‐positive status: 95.2% in our survey compared to 86.8% in the national survey [1]. Ensuring the availability of medicines and access to healthcare by abolishing user fees in the district in 2011 may have also convinced HIV positive people to seek treatment. Further advocacy for the initiation of ART at thresholds that were always higher than the recommended levels, and universal treatment of HIV was offered three months earlier in Gutu District than in the rest of the country. All these strategies may have played an important role in the ART coverage results of our study.

Although we found high results in the two first 90 targets of our survey, results in the third 90 were less promising. VLS among the ones on ART was still low, specifically among men, and half of all participants with a HIV‐RNA VL ≥ 1000 copies/mL were on ART. This suggests that one of the remaining critical gaps to prevent HIV mortality and combat the epidemic depends on achieving an increased quality of clinical care. This may reflect either poor adherence to ART that raises questions about adherence counselling, and HIV drug resistance. A recent article indicated that among people in Zimbabwe receiving ART for the first time, more than 10% carried a virus resistant to non‐nucleoside reverse transcriptase inhibitors [29]. This has serious implications, including an increased risk of mortality at the individual level and HIV transmission at the population level [15, 30, 31]. Better adherence, access to VL testing and use of new regimens (such as dolutegravir) could improve VLS among individuals on ART.

Overall, HIV incidence in Gutu District was similar to that at the national level when restricted to 15 to 64 year olds (0.43% vs. 0.47%) [1]. Incidence results in our survey were similar among men and women which differ from what has been observed in other surveys [13, 15, 25]. Low incidence may be explained by HIV‐testing strategies, health education and prevention strategies implemented in the district since 2011 as well as advocacy of Antiretroviral Treatment as Prevention (TaSP) [32] in 2015. If the district improves VLS among individuals on ART, it is expected that HIV incidence will likely continue to decrease in the coming years.

Strengths and limitations: Despite the age of the data, this study shows the importance of analysing the steps of the cascade of care and 90‐90‐90 targets in order to identify strengths and gaps in a programme to orient strategies to reduce HIV mortality, morbidity and new infections.

Moreover, the population‐based survey design minimised bias inherent to routinely available data. While findings at district level usually come from extrapolation or modelling studies, our results are generalizable to the entire population of Gutu District, including to HIV‐positive individuals who are not previously diagnosed. The inclusion rate was very high, even among men who had a higher rate in our study relative to other recent population‐based surveys [13, 14, 15]. It is possible that our recruitment strategy resulted in a better capture of men. Our HIV cascade of care and prevalence results may therefore be more accurate than other surveys; however, the differences observed may also have been due to heterogeneity across settings. In terms of limitation, most of the information collected was self‐reported which may have led to self‐reporting bias. Our study mitigated this potential bias in ART coverage, with blood level measurement of ART, which allowed for the reclassification of participants with positive ART blood levels who originally claimed to be unaware and not on ART. Similarly, overestimation of ART use was avoided through verification of the health booklet for participants self‐reporting being on ART.

Moreover, the cross‐sectional study design precludes drawing causal inferences from associations that were observed. As HIV rapid diagnostic test algorithms may sometimes lead to false results since ART reduces the sensitivity of HIV diagnosis, participants who tested HIV positive prior to the survey and were on ART might have had negative results during the onsite HIV test [33, 34, 35]. Therefore, the HIV prevalence might have been underestimated.

The use of the LC‐MS/MS assay to determine if participants were truly not on ART may have underestimated the ART coverage. Participants irregularly adherent to atanazavir (short half‐life) may have been negative to the LC‐MS/MS assay, and therefore classified as not on ART. Finally, as no baseline survey was conducted prior to the interventions in the district, we are unable to assess if the indicators have improved since the start of the intervention.

## 5 CONCLUSIONS

In conclusion, our study provides evidence that achievement of high results in HIV status awareness and ART coverage among HIV positive or among participants aware of their HIV status is feasible when utilizing a differentiated and decentralized model of care. However, the VLS among HIV positive or among participants on ART was relatively low. Quality care remains the most critical gap in the cascade of care in Gutu District to further reduce mortality and HIV transmission. Documentation and capitalization of experiences of differentiated provision of HIV services are needed in this setting to further understand those results.

## COMPETING INTERESTS

All authors declare that they have no conflicts of interest.

## AUTHORS’ CONTRIBUTIONS

NC, DBG, AM, TA, AP and DM developed the study protocol. ES led the data collection. ES, MC and NC participated in the data cleaning. NC and MC performed the laboratory and the statistical analysis. DM provided critical input in the laboratory and statistical analysis. AP provided critical revisions into the HIV incidence analysis and results. NC and RC drafted the article. EC provided critical revisions in the article, particularly in the discussion. All authors have read, reviewed and approved the final paper.
